# Job Satisfaction in Fisheries Compared

**DOI:** 10.1007/s11205-012-0059-z

**Published:** 2012-06-07

**Authors:** Richard Pollnac, Maarten Bavinck, Iris Monnereau

**Affiliations:** 1 University of Rhode Island, Kingston, RI USA; 2 Centre for Maritime Research (MARE), University of Amsterdam, Nieuwe Prinsengracht 130, Amsterdam, VZ 1018 The Netherlands

**Keywords:** Job satisfaction, Fishing, Asia, Africa, Caribbean fisheries management

## Abstract

This article draws comparative lessons from seven job satisfaction studies on marine capture fishing that were recently carried out in nine countries and three geographical regions—Asia, Africa, and the Caribbean. The seven studies made use of an identical job satisfaction assessment tool and present information on a selection of métiers mainly in the small-scale and semi-industrial fishing sectors. The responses manifest statistically significant geographical variation. Multidimensional plots and cluster analyses lead the authors to identify three clusters: (1) Southeast Asian (Vietnam and Thailand); (2) Caribbean (Belize, Nicaragua, Dominican Republic) and (3) Afro-Indian (Senegal, Guinea Bissau, and India). Jamaica is a significant outlier. On a general level, the authors conclude that fishers who report that they are not interested in leaving the occupation of fishing score higher on three traditional job satisfaction scales—basic needs, social needs and self actualization. Those who say they would leave fishing for another occupation are younger, have less fishing experience and smaller households. The latter findings are of relevance with regard to the pressing need, felt by fisheries managers, to move fishers out of the fishery.

## Introduction

This paper strives to draw comparative lessons from the seven job satisfaction studies from marine capture fishing presented in this special issue. The seven studies relate to three geographical regions—Asia, Africa, and the Caribbean—that play an important role in world fisheries today. The FAO estimates that these regions account for 84 % (FAO 2011) of global fish production. Moreover, these regions now include 97.7 % of those employed in the profession (FAO 2011). The studies in this special issue therefore provide information important to understanding the professional attitudes of a large segment of those employed in marine fisheries.

The nine countries in which job satisfaction surveys have been carried out are of widely divergent character. In Sect. 2 below, we compare the case study countries in terms of their overall development and the importance of their fisheries. Section 3 looks into similarities and differences between the fishing métiers that were studied. Together these sections provide the setting for a detailed analysis of the job satisfaction study results. Section 4 compares the demographic characteristics of the research populations, while Sect. 5 considers the responses to job satisfaction items. Section 6 then investigates the responses to three general questions, which ask into respondents’ attachment to the fishing profession. The latter is of relevance with regard to the pressing need, felt by fisheries managers, to move fishers out of the fishery (Daw et al. 2012). Section 7 provides concluding comments.

## Comparison of Case Study Countries

This section situates the set of job satisfaction studies in the context of their country environments. Table 1 presents an overview of country scores on the Human Development Index (HDI). These inform us of the level of poverty that prevails, and give an indication of the alternatives to fishing that may be available.

**Table 1:** Case study country scorings on human development index (source: UNDP 2011, accessed 14-12-11)

	Life expectancy (years)	Mean years of schooling	Multi-dimensional poverty index	Income Gini coefficient (inequality)	Composite HDI score	Rank
Belize	76.1	8.0	0.024	59.6	0.699	93
Dominican Republic	73.4	7.2	0.048	48.4	0.663	98
Guinea Bissau	48.1	2.3	n.a.	n.a.	0.353	176
India (Tamil Nadu)*	56.4 (66.7)	4.4 (n.a.)	0.283 (n.a.)	36.8 (n.a.)	0.547 (0.657)	134
Jamaica	73.1	9.6	n.a.	42.2	0.727	79
Nicaragua	74.0	5.8	0.128	52.3	0.589	129
Senegal	59.3	4.5	0.384	39.2	0.459	155
Thailand	74.1	6.6	0.006	53.6	0.682	103
Vietnam	75.2	5.5	0.084	37.6	0.593	128

* Data for Tamil Nadu are drawn from Government of Tamil Nadu 2003

The table demonstrates that while most of the case study countries are located in the middle ranges of HDI, three belong to the bottom third, suggesting underdevelopment. Guinea Bissau, and Senegal show particularly poor results on the HDI. Whereas India as a whole also scores low on HDI, the state of Tamil Nadu where the case study was situated has higher ratings.

## Comparison of Fishing Métiers

The seven job satisfaction studies present information on a selection of fishing métiers (i.e. a specific fishing activity determined by gear, area fished and target species). Some of these métiers focus on one species prevailing in local waters (such as shrimp, lobster, or sardines), while others—by nature of the gears employed—catch a wider range of species (see Table 2). Tropical fisheries are naturally more diversified than those in temperate waters, and a mixed species fishery often provides good opportunities for a stable livelihood. Generally speaking, the mixed species fisheries in our sample cater to the local market, whereas single species fisheries often—but not always—supply the high-value export market. The exception is the Senegal purse seine fishery, which caters to the domestic market. Fisheries in the developing world have been increasingly supplying fish to the global market (FAO 2005) and these studies are not an exception.

**Table 2:** Situation of case study métiers

	Sub-sector	Target species	Reason for importance	Major issues faced
Belize	Small-scale	Lobster	High value export earnings	Imposition MPA
Dominican Republic	Small-scale	Mixed	Large % fishing population/meet local food security needs. Lobster for export market	High level IUU fishing
Guinee Bissau	Small-scale	Mixed	Large % fishing population/meet local food security needs	Imposition MPA
India	Semi-industrial	Shrimp	Large contribution export earnings	Conflicts small-scale fisheries; declining stocks
Jamaica	Small-scale	Lobster	High value export earnings	High level IUU fishing
Nicaragua	Small-scale	Lobster	High value export earnings	High level IUU fishing
Senegal	Small-scale	Sardine	Large % fishing population/meet local food security needs	International fishing agreements EU
Thailand	Small-scale	Mixed	Large % fishing population/meet local food security needs	Declining stocks, illegal fishing
Vietnam	Semi-industrial	Shrimp	Large % fishing population/meet local food security needs	Imposition MPAs

Most of the métiers considered belong to the small-scale fishing sector, defined following common practice (see Johnson et al. 2005, 2006; Mills et al. 2011), as fishing that is labour intensive, technically simple, and of low cost. Our sample also includes two semi-industrial fisheries, India and Vietnam. These find their origin in the blue revolution that took place in many parts of the world in the second half of the twentieth century (Bavinck 2011). Both of these fisheries target shrimp. Although the number of people employed in the small-scale fisheries sector is generally larger, semi-industrial fisheries have grown rapidly in size and importance (Johnson 2006).

Table 2 situates the case study métiers and indicates their relevance for the countries concerned. Some métiers make a significant contribution to fisheries employment, and play an important role in meeting domestic food security needs. Others focus on high-value species, contributing in large measure to foreign exchange earnings. None of the investigated métiers is marginal to the countries concerned; all of them make a key contribution to their fisheries economies in one way or another.

Table 2 (column 5) also presents an inventory of major issues facing the case study fisheries, which affect the job satisfaction rates of the fishers involved. These are ecological, economic or social in nature and relate either to the fisheries or to the governing systems in place. Issues include the decline of fishing grounds, the rising price of fuel and declining prices of seafood products, the effects of the imposition of Marine Protected Areas (MPA), the prevalence of Illegal, Unreported and Unregulated (IUU) fisheries, and the continuation of fishery conflicts.

## Demographic Characteristics Compared

Social theory and other job satisfaction research suggest that job satisfaction is probably related to other social-demographic variables such as age, education, years and position in the occupation (e.g., crew vs. captain) and household size.

Table 3 indicates that as age increases so does satisfaction with the social needs component of job satisfaction. Age is not related to any of the other job satisfaction measures. In contrast, years of education is statistically significantly related to 3 of the job satisfaction measures: as education increases so does the level of satisfaction on basic needs and self actualization; conversely, as education increases satisfaction with management decreases. Years fishing is statistically significantly correlated with all three of the traditional job satisfaction components—as years in the occupation increase, so does satisfaction. Finally, the correlations in Table 3 indicate weak, but statistically significant relationships between family size and satisfaction with four of the job satisfaction measures. As household size increases, fishers are less satisfied with the basic needs, management and nature. Conversely, they are more satisfied with their level of self actualization. Table 4 indicates that single fishers score statistically significantly higher on all job satisfaction scales except satisfaction with management. With regard to crew status, crew members score statistically significantly higher with regard to satisfaction with management and lower on the nature scale.

**Table 3:** Correlations between selected social variables and job satisfaction scale scores

	Age	Years school	Years fishing	House-hold size
Basic needs	0.012	0.186**	0.160**	−0.155**
Social needs	0.108*	0.068	0.266**	0.015
Self actualization	0.051	0.216**	0.208**	0.133*
Management	−0.005	−0.205**	−0.065	−0.199**
Nature	0.018	−0.014	0.001	−0.120*

* *p* < 0.01 ** *p* < 0.001

**Table 4:** Job satisfaction scale scores by marital and crew status

	Marital status	Mean	*t* value	Crew status	Mean	*t* value
Basic needs	Single	*3.441*	5.307**	Crew	3.299	2.466
	Married	3.171		Captain	3.425	
Social needs	Single	*3.759*	3.045*	Crew	3.605	−2.021
	Married	3.599		Captain	3.713	
Self actualization	Single	*3.654*	4.975**	Crew	3.407	1.082
	Married	3.352		Captain	3.338	
Management	Single	3.325	1.435	Crew	*3.488*	3.216*
	Married	3.228		Captain	3.288	
Nature	Single	*3.272*	3.497*	Crew	3.056	3.533**
	Married	3.046		Captain	*3.299*	

* *p* < 0.01 ** *p* < 0.001. Italized values indicate highest value for statistically significant differences (*p* < 0.01, *t* test)

## Responses to Job Satisfaction Items Compared

In this section we examine the differences on the 5 job satisfaction components between the different countries. This was accomplished by conducting an analysis of variance across the 9 countries to determine if the between country variance in response exceeds the within country variance. The results of this analysis are in Table 5.

**Table 5:** Analysis of variance of cross country differences

Country	Basic needs	Social needs	Self-actualize	Manage	Nature
Belize	3.619	4.100	3.785	2.661	3.323
Jamaica	3.343	3.508	3.103	2.974	3.769
Nicaragua	3.570	3.883	3.526	3.276	3.577
Dominican Rep.	3.666	3.940	3.803	3.586	3.335
Guinea Bissau	2.987	3.548	3.667	2.957	3.090
India	3.378	3.710	3.493	3.245	2.853
Senegal	2.778	3.701	3.756	2.507	2.838
Vietnam	2.753	3.177	2.775	3.816	2.851
Thailand	3.167	3.250	2.811	3.450	3.350
*F* ratio	38.289	21.066	33.618	29.309	12.717
d.f.*	8.596	8.594	8.605	8.599	8.607
*p*	<0.001	<0.001	<0.001	<0.001	<0.001

* N varies due to missing data

**Fig. 1:**
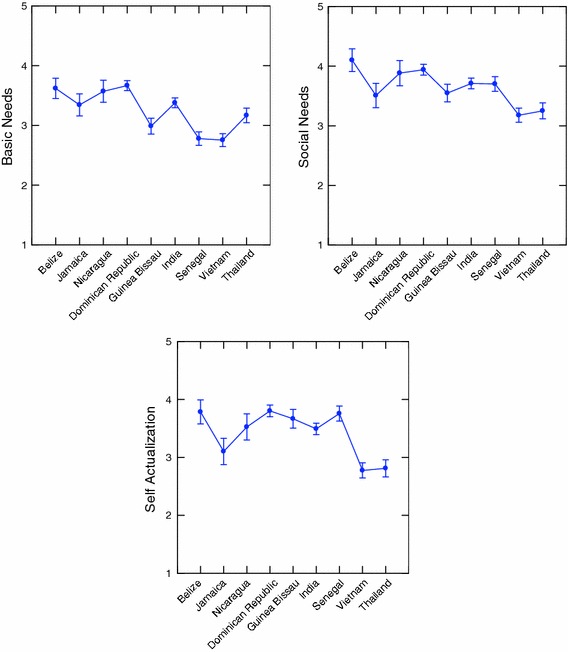
Mean values for traditional job satisfaction scales

**Fig. 2:**
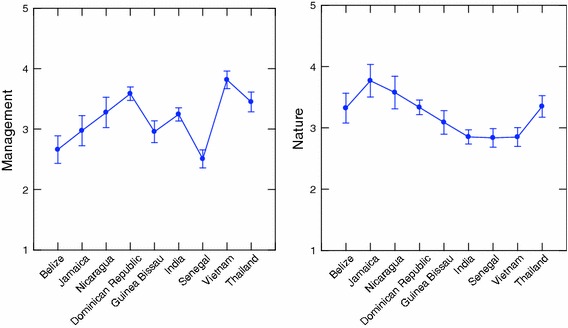
Mean values for management and nature job satisfaction scales

**Fig. 3:**
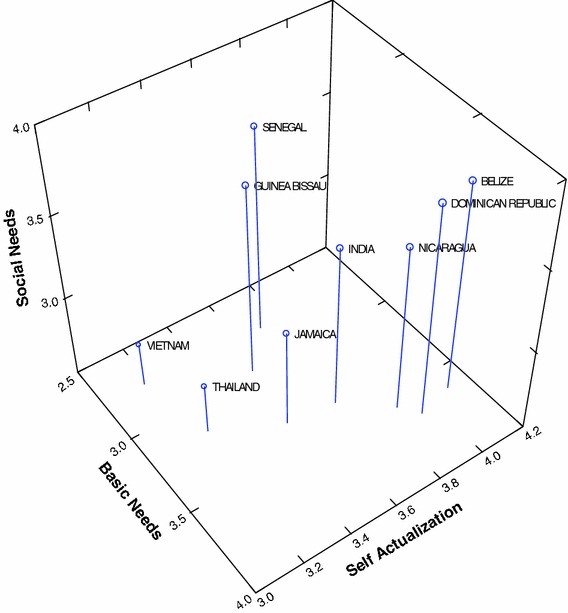
Traditional job satisfaction scales for the 9 countries plotted in 3 dimensional space

**Fig. 4:**
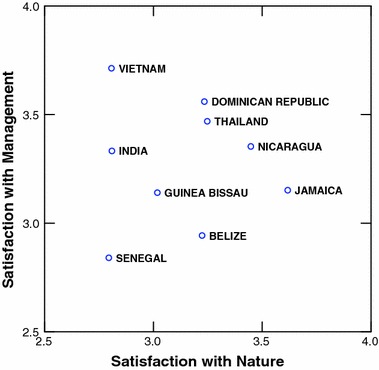
Mean values for management and nature job satisfaction scales for the 9 countries plotted in 2 dimensional space

Table 5 clearly indicates that for all 5 job satisfaction scales, the between country variance exceeds the within country variance. All differences are statistically significant (*p* < 0.001). These values are plotted in Figs. 1, 2, 3, 4. Figures 1 and 2 illustrate differences between the countries on each variable separately. Figures 3 and 4 best illustrate the differences between the 9 countries with respect to scores on the job satisfaction scales. For example in Fig. 3, in 3-dimensional job satisfaction space, it can be clearly seen that Vietnam scores lowest on all 3 dimensions while Belize and the Dominican Republic score quite high on all 3. In Fig. 4 Senegal is in the lowest quarter of the management and nature scales, and the Dominican Republic, Thailand and Nicaragua are in the highest quarter. Vietnam scores highest with regard to satisfaction with management but is about as low as Senegal and India with regard to nature. Although Senegalese fishers are score medium on self-actualization they show very low on all other categories. Viewing the countries in multidimensional space provides a better illustration of their overall similarities and differences.

**Fig. 5:**
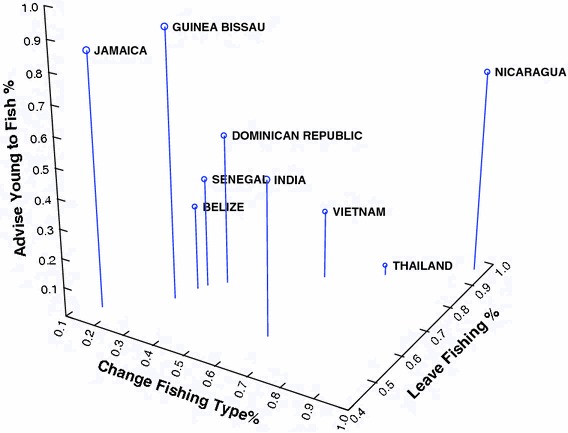
Percent “yes” responses to the three job satisfaction questions plotted in three dimensional space

Table 6 presents percent distribution of “yes” responses to the 3 job satisfaction questions concerning whether the fisher would change fishing type, leave the occupation of fishing for another occupation, or advise a young person to enter the occupation. A Chi-square analysis of the differences across the 9 countries indicates that all differences are statistically significant (*p* < 0.001). The results of this analysis are plotted in 3 dimensional space in Fig. 5. Focusing on Fig. 5, Jamaica and Guinea Bissau appear close together in the 3-dimensional space—both are relatively high with regard to advising a young man to fish, low on willingness to change fishing type and moderate on willingness to change occupation. Senegal, Belize and the Dominican Republic appear as another cluster being similar with regard to percent willing to change occupation and fishing type, but manifesting an increasing percent of willingness to advise a young person to enter the occupation as one moves from Senegal through Belize to the Dominican Republic in the 3-dimensional plot. In this same job satisfaction space, Thailand, Vietnam and Nicaragua—although individually different—stand out from the others being relatively high on willingness to change both fishing type and occupation.

**Table 6:** Percent variation in “yes” responses to job satisfaction questions

Country	Change type	Leave fishing	Advise young
Belize	32.6	61.3	29.0
Jamaica	15.4	46.2	84.6
Nicaragua	96.2	96.2	69.2
Dominican Rep.	37.7	66.2	51.5
Guinea Bissau	30.0	56.0	90.0
India	63.7	53.5	51.5
Senegal	33.8	63.3	37.5
Vietnam	60.3	78.0	23.6
Thailand	75.0	85.0	3.3
χ^2^	91.067	38.446	124.201
*d.f.*	8	8	8
Cramer’s V	0.386	0.259	0.459
*p*	<0.001	<0.001	<0.001

Tables 5 and 6 and Fig. 1, 2, 3, 4, 5 illustrate the complexity of the distribution of values on the 8 different job satisfaction indicators across the small-scale fishermen examined in the 9 nations. Senegal, Guinea Bissau and India seem to cluster together in Figs. 4 and 5, with Guinea Bissau’s high value on willingness to advise a young man to fish separating it a bit from the other two in Fig. 5. Another repeating pattern is found with regard to Thailand and Vietnam, which are never very far apart in Figs. 2, 3, 4. Finally, the Dominican Republic, Belize and Nicaragua are clustered in Fig. 2, Belize moves from this cluster in Fig. 4 and Nicaragua is more removed in Fig. 4, but at least 2 out of the three are in relative close proximity in all 3 multidimensional figures.

**Fig. 6:**
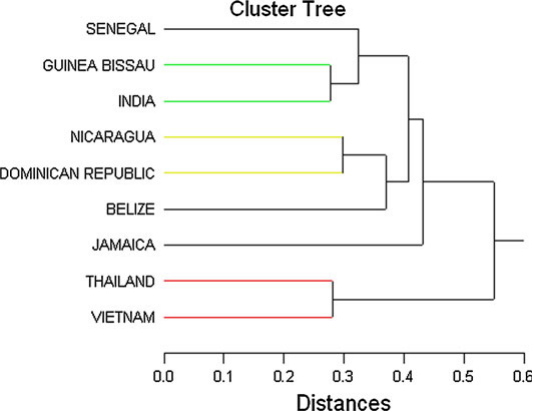
Hierarchical cluster analysis (Euclidian distance, average link method) of nations based on 8 job satisfaction variables

These complex relationships might be best depicted if we used an analytic technique that could determine relative similarities and differences between the 9 nations on the 8 indicators of job satisfaction simultaneously. The appropriate technique for this task is hierarchical cluster analysis. The form of hierarchical cluster analysis used here initially treats each nation as a separate cluster. It then joins clusters in a stepwise manner based on a distance metric; in this analysis we used normalized Euclidean distance (root mean squared distances). First, the two closest nations are combined into a new cluster and average distances of the included nations with each other nation are calculated. The process continues in a stepwise manner, combining nations (or clusters of nations) until all nations are included in one cluster. Figure 6 presents the results of this hierarchical cluster analysis.

The analysis represented in Fig. 6 indicates that Vietnam and Thailand form the “tightest” cluster. The next tightest clusters are composed of Guinea Bissau and India, followed by Nicaragua and the Dominican Republic. Tightness of nations or clusters is indicated by the distance in Fig. 6—the smaller the distance of linking, the tighter the cluster. Senegal is next joined with the Guinea Bissau and India cluster, followed by the joining (at a greater distance) of Belize into the Nicaragua and the Dominican Republic cluster. The distance of Jamaica is too great to be considered a member of any of the clusters. The analysis also indicates that the Senegal, Guinea Bissau, India (Afro-Indian Cluster) and the Belize, Nicaragua, Dominican Republic clusters (Caribbean Cluster) are closer to each other than they are to the Vietnam, Thailand cluster (Southeast Asian Cluster). Geographically and culturally these clusters seem to make sense. The only anomaly is Jamaica, which we cannot explain.

## Attachment to the Fishing Profession

Turning to the 3 job satisfaction questions, Table 7 indicates that those who are willing to change fishing type have statistically significantly less education and fishing experience. Those who say they would leave fishing for another occupation are younger, have less fishing experience and smaller households. Household size is also statistically significantly related to advising a young person to become a fisher—the larger the household, the more likely to advise a young person to fish. Finally, those who are married and those who are captains are more likely to want to change their fishing type (Tables 8 and 9).

**Table 7:** Difference in age, education, years fishing and household size by response to job satisfaction questions

	Mean value					
Variable	Change fishing type	*t*	Leave fishing	*t*	Advise young to fish	*t*
Age						
No	40.667	1.459	*41.367*	2.145*	39.288	–1.127
Yes	39.248		39.097		40.405	
Years education						
No	*5.886*	2.246*	5.563	−0.283	5.400	–1.315
Yes	5.147		5.663		5.839	
Years fishing						
No	*20.982*	2.191*	*23.193*	3.998***	19.129	–1.848
Yes	18.803		18.869		21.019	
Household size						
No	6.026	1.292	*6.761*	2.802**	5.229	–2.687**
Yes	5.454		5.396		*6.322*	

* *p* < 0.05 ** *p* < 0.01 *** *p* < 0.001. Italized values indicate highest value for statistically significant differences (*p* < 0.05, *t* test)

**Table 8:** Percent variation in “yes” responses to job satisfaction questions

Marital status	Change type	Leave fishing	Advise young
Single	39.9	64.3	50.5
Married	54.6	65.5	44.4
χ^2^	11.070	0.074	1.921
*d.f.*	1	1	1
Phi	0.135	0.011	−0.057
*p*	<0.01	>0.01	>0.01

**Table 9:** Percent variation in “yes” responses to job satisfaction questions

Crew status	Change type	Leave fishing	Advise young
Crew	47.8	65.2	47.8
Captain	63.6	67.6	37.7
χ^2^	11.970	0.280	4.774
*d.f.*	1	1	1
Phi	0.158	0.025	−0.102
*p*	<0.01	>0.01	>0.01

Table 10 examines relationships between the three traditional job satisfaction components and the satisfaction with nature and management scales. Here we find that satisfaction with nature is statistically significantly related to both the basic and social needs components—as satisfaction with nature increases so does the level of satisfaction on the two components. Finally, the self actualization scale manifests a statistically significant, but relatively weak negative correlation with satisfaction with management—the more one is satisfied with management, the less they are satisfied with their level of self actualization.

**Table 10:** Correlations between selected job satisfaction scales

	Management	Nature
Basic needs	0.054	0.268**
Social needs	−0.052	0.127*
Self actualization	−0.130*	0.045

* *p* < 0.01 ** *p* < 0.001

Finally Table 11 presents the relationships between the 3 job satisfaction questions and the 5 job satisfaction scales. Looking only at the statistically significant differences, we see that those who say they will *not* change fishing type score higher on the social needs and self actualization scales. Fishers who report that they are *not* interested in leaving the occupation of fishing score higher on the 3 traditional job satisfaction scales—basic needs, social needs and self actualization. Finally, those who *would* advise a young person to become a fisher, score higher on all except the management scale, where there is no statistically significant difference. All of these relationships between the 3 questions and scores on the 5 job satisfaction scales are in the expected direction—high scores result in responses reflecting a positive attitude towards the occupation of fishing.

**Table 11:** Difference of mean scores on job satisfaction scales by response to job satisfaction questions

Variable	Mean scale scores					
	Change fishing type	*t*	Leave fishing	*t*	Advise young to fish	*t*
Basic needs						
No	3.244	−0.722	*3.397*	2.902*	3.177	4.825**
Yes	3.278		3.249		*3.407*	
Social needs						
No	*3.719*	2.780*	*3.823*	3.806**	3.588	3.976**
Yes	3.585		3.632		*3.778*	
Self actualization						
No	*3.541*	3.176*	*3.693*	5.520**	3.358	4.211**
Yes	3.362		3.362		*3.598*	
Management						
No	3.251	−0.031	3.154	−0.729	3.256	1.345
Yes	3.252		3.200		3.174	
Nature						
No	3.188	2.401	3.197	1.171	3.011	4.325**
Yes	3.044		3.121		*3.274*	

* *p* < 0.01 ** *p* < 0.001. Italized values indicate highest value for statistically significant differences (*p* < 0.01, *t* test)

## Conclusion

All 5 job satisfaction scales and the responses to the 3 job satisfaction questions manifest statistically significant variation across the 9 countries. Multidimensional plots and cluster analyses led us to conclude that while Jamaica seems quite different from the others, the remaining nine can be gathered into 3 clusters: (1) Southeast Asian (Vietnam and Thailand); (2) Caribbean (Belize, Nicaragua, Dominican Republic) and (3) Afro-Indian (Senegal, Guinea Bissau, and India). The results between the cluster Southeast-Asian and Afro-Indian are striking. The countries in the first cluster are emerging economies (Table 1), in which alternative employment to fishing is probably more readily available. This could explain the high scores on willingness to leave fishing, and low scores in advising a young person to fish. The second cluster consists of countries low on HDI, in which alternative employment is less prevalent and fishers are more likely to want to maintain a stake in the profession. Daw et al. (2012), studying job satisfaction among small-scale fishers in five western Indian Ocean countries, does not find evidence of a relationship between economic development and higher readiness to exit, instead citing site-specific characteristics.

The relationships between social-demographic variables and the various job satisfaction measures seem to make sense. The positive correlations between satisfaction with Basic Needs and years of schooling and experience in the occupation can be interpreted as indicating that better educated and more experienced fishers will probably be both more productive and fish more safely. The positive relationship with a fishers’ single status and negative relationship with household size can probably be explained by the assumption that single fishers and those with smaller households will have less demands on their income; therefore, will not need as much to be satisfied. As workers, in this instance fishers, gain more experience with life and the requirements of their occupation adjustments to improve social relationships would probably be accomplished. This could account for the positive relationships observed between satisfaction with Social Needs and age and years fishing experience. The life of a non-married fisher would require fewer adjustments than a married fisher, probably explaining the positive relationship between single status and satisfaction with Social Needs.

Correlates of satisfaction with Self Actualization Needs are more difficult to explain. Here our interpretations are more conjectural. Does more formal education provide the fisher with enough worldly knowledge to make positive comparisons concerning the independence, challenge and adventure of fishing? Is it true that the longer one fishes, the more likely they are to recognize these attributes of fishing? Does the contrast between a large, noisy and demanding household and the challenge and adventure of the sea provide the fisher with a real appreciation for these aspects of his occupation? And, paradoxically, are single men more likely to appreciate the challenges and adventures of fishing? Clearly, these relationships require more research.

Those with more fishing experience and with captain status are probably less satisfied with management because they feel that their status and experience are being challenged by bureaucrats who know nothing about fishing—statements actually made by East Coast fishers in the USA concerning management. The negative correlation with household size may be the result of those from larger households, with greater needs, may feel that management restrictions will reduce income and their ability to support their family. The fact that fishers from larger households are less satisfied with Nature Needs might also reflect greater needs for sustaining a larger household which make them view the ability of their environment to support their household with a more critical eye. It is difficult to explain why those who are single and work as crew members would be less satisfied with the items on the Nature Needs scale.

Finally, turning to correlates of the 3 job satisfaction questions we find that those who say they would change type of fishing have less formal education and fishing experience. Perhaps they are less able to evaluate the advantages of their current métier in contrast to others. The finding that younger fishers and those with less fishing experience are more likely to say they would leave fishing if given the opportunity is probably related to the fact that they have less time invested in the occupation. The fact that those with larger households are less likely to leave suggests that with the greater responsibility to support more family, they are afraid to take the chances associated with a complete change in occupation.

Other variables expected to influence one’s loyalty to one’s occupation are the satisfactions derived from it. It is therefore reasonable to expect that satisfaction on the 5 components of job satisfaction examined here would influence a fisher’s responses to the 3 job satisfaction questions. Above we found that those who say they will *not* change fishing type score higher on the social needs and self actualization scales. More importantly, we found that fishers who report that they are *not* interested in leaving the occupation of fishing score higher on the 3 traditional job satisfaction scales—basic needs, social needs and self actualization. Finally, we found that those who *would* advise a young person to become a fisher score higher on all except the management scale, where there is no statistically significant difference. All of these relationships between the 3 questions and scores on the 5 job satisfaction scales are in the expected direction—high scores result in responses reflecting a positive attitude towards the occupation of fishing. And it makes sense that those with a positive attitude towards their occupation would resist change. This is an extremely important finding which can help explain why attempts to reduce pressure on the resource by reducing numbers of fishermen frequently result in failure.

Most significant is the finding that fishers not willing to change occupation are likely to score high on the Self Actualization Scale and have more years of fishing experience. This finding supports other claims that Self Actualization is an important explanatory factor concerning the apparently irrational resistance of fishers to pressures and incentives for leaving the occupation of fishing (Pollnac et al. 2006; Pollnac and Poggie 2008; Pollnac 2011). Attempts to “rationalize” the fishery by reducing numbers of boats and fishermen need to account for the impacts this psychological variable can have on fishers’ lives, families and communities to either develop appropriate alternative occupations for displaced fishers or prepare for the resulting negative impacts.
